# Postoperative enterocolitis assessment using two different cut-off values in the HAEC score in Hirschsprung patients undergoing Duhamel and Soave pull-through

**DOI:** 10.1186/s12887-020-02360-x

**Published:** 2020-10-02

**Authors:** Gunadi, Afnandito Valeno Risky Sukarelawanto, Azmi Ritana, Naisya Balela, Wayan Julita Krisnanti Putri, Dian Nirmala Sirait, Vincentia Meta Widya Paramita, Andika Purba Sasmita, Andi Dwihantoro, Akhmad Makhmudi

**Affiliations:** Pediatric Surgery Division, Department of Surgery, Faculty of Medicine, Public Health and Nursing, Universitas Gadjah Mada/Dr. Sardjito Hospital, Jl. Kesehatan No. 1, Yogyakarta, 55281 Indonesia

**Keywords:** Duhamel procedure, Gestational age, Hirschsprung disease, HAEC, Risk factors, Mothers’ age at childbirth, Mothers’ educational level, Sex, Soave pull-through

## Abstract

**Background:**

Hirschsprung-associated enterocolitis (HAEC) is the most severe and potentially lethal complication of Hirschsprung disease (HSCR) which might occur following definitive surgery. Our objectives were: 1) to compare the incidence of HAEC after Duhamel and Soave procedures using different cut-off values of the HAEC scoring method; and 2) to associate them with the risk factors, including sex, aganglionosis type, mothers’ age at childbirth, gestational age, and mothers’ educational level.

**Methods:**

Medical records of patients with HSCR who underwent Soave and Duhamel procedures in our institution, Indonesia (January 2012 – December 2016) were reviewed retrospectively. Two cut-off values of the HAEC scoring system (i.e.*,* ≥10 and ≥ 4) were utilized.

**Results:**

Eighty-three patients with HSCR were recruited in this study (Soave: 37 males and 7 females vs. Duhamel: 28 males and 11 females; *p* = 0.18). The incidence of HAEC after surgery was 14/83 (16.9%) and 38/83 (45.8%) for cut-off values of ≥10 and ≥ 4, respectively (*p* = 0.00012), and tended to have an association with sex (*p* = 0.09). Although it was not statistically significant (*p* = 0.07), the frequency of HAEC after Soave procedure tended to be higher in patients with their mother’s age of ≤35 years at childbirth than those with their mother’s age of > 35 years (OR = 7.9; 95% CI = 0.9–72.1). Multivariate analysis indicated none of the risk factors were associated with the frequency of HAEC after definitive surgery.

**Conclusions:**

The lower cut-off value of ≥4 might increase the possibility to diagnose HAEC, particularly the mild cases. The incidence of HAEC after definitive surgery was not associated with any risk factors in our cohort patients. Further multicenter studies with a larger sample size are necessary to confirm our findings.

## Background

Hirschsprung disease (HSCR) is characterized by the absence of ganglion cells in the intestines, which results in a functional obstruction in children [1]. Its incidence among specific ethnic groups is reported as 1.5/10,000, 2.1/10,000, and 2.8/10,000 live births in Europeans, African Americans and Asians, respectively [2]. These data might correspond to a recent study confirming that Indonesian controls show higher frequency of *RET* rs2435357 and rs2506030 risk alleles compared to other ethnic groups [3, 4].

The treatment of choice for HSCR is resection of the aganglionic colon segment and anastomosis of a normally ganglionated bowel section to the anus. There are various modifications of pull-through surgery for HSCR, including the Duhamel and Soave procedures [1].

Complications of HSCR include Hirschsprung-associated enterocolitis (HAEC) [5]. HAEC may happen pre- or post pull-through surgery [6–8]. The incidence of preoperative and postoperative HAEC varies among studies [7–10]. Several risk factors have been associated with HAEC, however, the findings are still controversial [7, 10–13]. Moreover, several scoring systems have been proposed to diagnose HAEC [14–16]. Our objectives were: 1) to compare the HAEC incidence after Duhamel and Soave procedures using different cut-off values of the HAEC scoring method; and 2) to associate them with the risk factors, including mothers’ age at childbirth, gestational age, and mothers’ educational level.

## Methods

### Patients

This retrospective study included all patients with HSCR who underwent Soave and Duhamel procedures at our institution (January 2012 – December 2016).

In this study, 83 subjects were eligible for the study (Duhamel = 39 vs. Soave = 44). Out of the 39 patients post Duhamel procedure, there were 28 male patients and 11 female patients. Patients post Soave procedure consisted of 37 male patients and 7 female patients. This research was approved by the Medical and Health Research Ethics Committee of the Faculty of Medicine, Public Health and Nursing, Universitas Gadjah Mada/Dr. Sardjito Hospital (KE/FK/787/EC/2015 and KE/FK/1356/EC/2015).

### Duhamel and soave procedures

Duhamel and Soave procedures were chosen based on the discretion of the attending physician and performed according to a previous report [10].

### HAEC scoring systems and risk factors

The HAEC scoring methods were used to diagnose HAEC [14–16]. Pastor et al. [14] introduced the cut-off value of 10 to diagnose HAEC, while others proposed the cut-off value of 4 to determine HAEC [15, 16]. In this study, we compared both cut-off values.

The risk factors included gestational age, maternal age at childbirth, and maternal educational level. We classified the mothers’ age at childbirth, gestational age and maternal educational level into ≤35 and > 35 years, preterm and full-term, and no education – elementary and junior, high school – bachelor, respectively.

The frequency of HSCR patients with Down syndrome in this cohort was 5/39 (12.8%) and 5/44 (11.4%) in the Duhamel and Soave groups, respectively.

### Statistical analysis

Data were presented as frequency or median (interquartile range, IQR). While Chi-square or Fisher Exact tests were used for analyzing the significant differences and the association between nominal variables, Mann-Whitney test was utilized for evaluating the significant differences between non-normal distribution variables. Multivariate logistic regression analysis was performed using *p*-value < 0.05 considered as significant with 95% confidence interval (CI).

## Results

### Incidence of HAEC after pull-through

There were 39 and 44 HSCR patients who underwent Duhamel and Soave procedures, respectively, with most patients (79.5%) were found with short-segment aganglionosis. No significant differences in the baseline characteristics between groups were noted (*p* > 0.05) (Table 1).

**Table 1:** HSCR patients’ characteristics after definitive surgery

Characteristics	Duhamel (n, %; median, IQR)	Soave (n, %; median, IQR)	***p***-value
Gender			0.17*
■ Male	28/39 (71.8)	37/44 (84.1)	
■ Female	11/39 (28.2)	7/44 (15.9)	
Aganglionosis type			0.28*
■ Short-segment	33/39 (84.6)	33/44 (75)	
■ Long-segment	6/39 (15.4)	11/44 (25)	
Age at HSCR diagnosis (months)	18.5 (5–60)	23 (9–64)	0.99**
Age at pull-through (months)	57 (47.3–82.8)	25 (13.4–70.5)	0.56**

*, *p*-values were calculated using Chi-square test; **, *p*-values were calculated using Mann-Whitney test; HSCR, Hirschsprung disease

None of the HAEC score findings were different between the two cut-off groups, except history of enterocolitis, explosive discharge of gas and stool on rectal examination, and multiple air fluid levels with *p*-values of 0.03, 0.02, and 0.04, respectively (Table 2).

**Table 2:** Comparison of HAEC findings in HSCR patients after pull-through between two cut-off values of the HAEC scoring system

HAEC Findings	Cut-off ≥ 10	Cut-off ≥ 4	*p*-value
History			
■ Diarrhea with explosive stool	8/14	12/38	0.09
■ Diarrhea with foul-smelling stool	10/14	16/38	0.12
■ Diarrhea with bloody stool	4/14	5/38	0.23
■ History of enterocolitis	10/14	14/38	0.03*
Physical examination			
■ Explosive discharge of gas and stool on rectal examination	9/14	11/38	0.02*
■ Distended abdomen	13/14	29/38	0.25
■ Decreased peripheral perfusion	2/14	5/38	1.00
■ Lethargy	10/14	25/38	0.75
■ Fever	11/14	28/38	1.00
Radiologic examination			
■ Multiple air fluid levels	7/14	7/38	0.04*
■ Dilated loops of bowel	7/14	9/38	0.09
■ Sawtooth appearance with irregular mucosal lining	2/14	2/38	0.56
■ Cut-off sign in rectosigmoid with absence of distal air	5/14	5/38	0.11
■ Pneumatosis	1/14	1/38	1.00
Laboratory			
■ Leukocytosis	9/14	24/38	1.00
■ Shift to left	8/14	20/38	1.00

*, *p*-values were calculated using Fisher Exact or Chi-square tests and *p* < 0.05 was considered significant; HAEC, Hirschsprung-associated enterocolitis

### Risk factors for HAEC after definitive surgery

Firstly, we compared the association between risk factors (i.e. sex and aganglionosis type) and incidence of HAEC in all HSCR patients after both pull-through procedures using two different cut-off values (Table 3). The incidence of HAEC after both pull-through was 14/83 (16.9%) and 38/83 (45.8%) for cut-off of ≥10 and ≥ 4, respectively (*p* = 0.00012).

**Table 3:** Comparison of HAEC incidence after pull-through between two cut-off values of HAEC scoring system and its association with patients’ characteristics

	Sex		***p***-value	OR (95% CI)	Aganglionosis type		***p***-value	OR (95% CI)
	Male	Female			Short-segment	Long-segment		
Cut-off ≥10 HAEC (n, %)	12/14 (86)	2/14 (14)	0.47	1.8 (0.4–9.0)	12/14 (86)	2/14 (14)	0.53	1.7 (0.3–8.3)
Cut-off ≥4 HAEC (n, %)	33/38 (87)	5/38 (13)	0.09	2.9 (0.9–8.4)	30/38 (79)	8/38 (21)	0.91	0.9 (0.3–2.7)

*, *p*-values were calculated using Fisher Exact or Chi-square tests; CI, confidence interval; HAEC, Hirschsprung-associated enterocolitis; OR, odds ratio

Male patients tended to have higher incidence of HAEC than female patients (*p* = 0.09; OR = 2.9 (95% CI = 0.9–8.4) (Table 3).

Subsequently, we determined the association between risk factors (i.e. sex and aganglionosis type) and incidence of HAEC after each surgical procedure using two different cut-off values of ≥4 (Table 4) and ≥ 10 (Supplement Table 1). There were 16 (41%) patients with HSCR after Duhamel surgery diagnosed with HAEC, while 22 (50%) patients post Soave procedure suffered from HAEC (*p* = 0.41). In addition, there were no associations of gender nor aganglionosis type with HAEC frequency after Duhamel (*p* = 0.28 and 0.63, respectively) and Soave procedures (*p* = 0.23 and 0.73, respectively) (Table 4). Also, no associations between patients’ characteristics and HAEC following Duhamel and Soave procedures were noted using cut-off value of ≥10 (Supplement Table 1).

**Table 4:** Association between patients’ characteristics and HAEC (cut-off ≥ 4) after Duhamel and Soave surgeries

	Sex		***p***-value	OR (95% CI)	Aganglionosis type		***p***-value	OR (95% CI)
	Male	Female			Short-segment	Long-segment		
Duhamel HAEC (n, %)	13/16 (81)	3/16 (19)	0.28	2.3 (0.5–10.6)	13/16 (81)	3/16 (19)	0.63	0.7 (0.1–3.7)
Soave HAEC (n, %)	20/22 (91)	2/22 (9)	0.23	2.9 (0.5–17.1)	17/22 (77)	5/22 (23)	0.73	1.3 (0.3–5.0)

*, *p*-values were calculated using Fisher Exact or Chi-square tests; CI, confidence interval; HAEC, Hirschsprung-associated enterocolitis; OR, odds ratio

Next, we determined the association between mother’s age at childbirth, gestational age, and mother’s educational level and HAEC after pull-through using cut-off of 4. Although it was not statistically significant (*p* = 0.07), the frequency of HAEC after Soave procedure tended to be higher in patients with their mother’s age of ≤35 years at childbirth than those with mother’s age of > 35 years (OR = 7.9; 95% CI = 0.9–72.1) when we analyzed them using cut-off value of 4 (Table 5). Moreover, there were no significant associations between mother’s age at childbirth, gestational age, and mother’s educational level with development of HAEC after Duhamel pull-through with *p*-values of 0.17, 0.36, and 0.59, respectively (Table 5). None of the maternal risk factors significantly affected the incidence of HAEC (cut-off ≥10) after Duhamel and Soave pull-through procedures (Supplement Table 2).

**Table 5:** Risk factors for HAEC (cut-off ≥4) following Duhamel and Soave surgeries

	Mothers’ age at childbirth (years)		p*	OR (95% CI)	Gestational age		p*	OR (95% CI)	Maternal educational level		p*	OR (95% CI)
	≤35	> 35			Preterm	Full-term			No education-elementary	Junior, high school - bachelor		
Duhamel HAEC (n, %)	10/16 (63)	6/16 (37)	0.17	0.4 (0.1–1.5)	1/16 (6)	15/16 (94)	0.36	4.5 (0.2–119)	3/16 (19)	13/16 (81)	0.59	0.7 (0.1–3.1)
Soave HAEC (n, %)	21/22 (95)	1/22 (5)	0.07	7.9 (0.9–72.1)	1/22 (5)	21/22 (95)	0.56	0.5 (0.04–5.7)	5/22 (23)	17/22 (77)	0.12	0.4 (0.1–1.3)

*, *p*-values was calculated using Fisher-Exact test; HAEC, Hirschsprung-associated enterocolitis; OR, odds ratio; CI, confidence interval

### Multivariate analysis

Then, we conducted a multivariate logistic regression analysis which showed none of risk factors were associated with the frequency of HAEC after definitive surgery (Table 6). Moreover, none of risk factors had any significant association with the incidence of HAEC (cut-off ≥10) after pull-through (Supplement of Table 3).

**Table 6:** Logistic regression of risk factors and HAEC (cut-off ≥4) after pull-through in our institution

	Sex		Aganglionosis type		Mother’s age at childbirth		Gestational age		Maternal educational level	
	p*	OR (95% CI)	p*	OR (95% CI)	p*	OR (95% CI)	p*	OR (95% CI)	p*	OR (95% CI)
Duhamel	0.14	0.3 (0.04–1.5)	0.76	1.4 (0.2–9.3)	0.12	3.7 (0.7–19.0)	1.00	–	0.99	1.0 (0.2–5.5)
Soave	0.18	0.3 (0.04–1.8)	0.99	1.0 (0.2–5.0)	0.08	0.1 (0.01–1.3)	0.54	2.3 (0.2–31.8)	0.17	2.7 (0.7–11.1)

*, *p*-values were calculated using logistic regression test

## Discussion

Here, we show the frequency of HAEC after definitive surgery was significantly higher (~ 3-fold) using the HAEC scoring system with cut-off ≥4 than ≥10. It has been proposed that a reduced cut-off of the HAEC scoring method to 4 can increase the sensitivity (83.72%) and specificity (98.63%) of HAEC diagnosis [15, 16]. In addition, if the cut-off of the HAEC scoring method changes into 2, the sensitivity to establish the HAEC diagnosis is even higher (86.05%), but it has lower specificity (95.89%) [15]. Our findings add another evidence that use of cut-off value of ≥4 might increase the number of diagnoses of HAEC, especially the mild cases. There are several novelties in our study: 1) we compared the cut-off values of ≥4 and ≥ 10 to diagnose HAEC between transabdominal Soave and Duhamel pull-through (vs. all procedures [Soave, Swenson and transanal- pull-through] as one group [16]; 2) we associated several risk factors and HAEC frequency after pull-through (vs. HAEC frequency preoperatively [7]); and 3) we performed the study on Indonesian patients with HSCR (vs. Caucasian population [15, 16]). It has been hypothesized that patients with specific genetic variants might have a higher possibility for HAEC occurrence [17]. Although HSCR patients with *NOD2* variants did not show any HAEC episode [18], interestingly, 59% of HSCR patients with *ITGB2* mutations suffered severe HAEC [19]. Further studies with different populations are necessary to confirm the association between genetic variants and HAEC frequency.

Most of our patients with HAEC (cut-off ≥4) had distended abdomen (76.3%), fever (73.4%), lethargy (65.8%), and leukocytosis (63.2) (Table 2). Only a few HAEC patients showed sawtooth appearance with irregular mucosal lining (2/38, 5.3%) and pneumatosis (1/38, 2.6%) (Table 2) in our cohort study, which is comparable with a recent report (1/43 [2.3%] and 1/43 [2.3%], respectively) [15]. Frykman et al. [15] suggested a new HAEC scoring system using four variables: diarrhea with explosive stool, decreased peripheral perfusion, lethargy, and dilated loops of bowel since all of those variables were strong predictors for HAEC. However, the history of enterocolitis, explosive discharge of gas and stool on rectal examination, and multiple air fluid levels had a significant frequency difference between the two cut-off values in our study (Table 2). Therefore, it is interesting to further clarify which findings within the HAEC scoring system that can be used as significant predictors for HAEC occurrence.

There are many risk factors affecting the development of HAEC, including Down syndrome (trisomy 21) [20]. Furthermore, mothers’ age at childbirth is associated with Down syndrome risk for their infant [21, 22]. Although not statistically significant, our study revealed that the risk of HAEC after Soave procedure tended to increase (~ 8-fold) in infants with their mother’s age of ≤35 years at childbirth. There are no particular studies that directly associate the mother’s age at childbirth and HAEC incidence after pull through, but there are some studies that associated the maternal age with HSCR frequency [23, 24]. HSCR was more common in infants with mothers’ age of ≥30 years at childbirth [23]. However, Löf Granström et al. [24] did not find any significant association between mothers’ age and HSCR frequency.

There is no evidence supporting the direct connection between the occurrence of HAEC after pull-through and gestational age. Moreover, our study failed to prove the gestational age as a risk factor for HAEC after pull-through.

In this study, the maternal educational level did not appear to affect the occurrence of HAEC after Duhamel and Soave procedures. We assumed that well-educated mothers will be more aware regarding any health problems during pregnancy and the health of their progeny. Therefore, educational status is essential. Besides, maternal education regarding prenatal screening is important because less awareness toward prenatal screening will increase the risk of having an infant with Down syndrome [25, 26]. Since Down syndrome increases the risk of HAEC, parents should be more aware about the disease if they have children with Down syndrome [12, 25, 27].

Long-segment aganglionosis has been related with higher risk of HAEC [11]. However, we did not find any association between type of aganglionosis and HAEC frequency, which is compatible with a recent study [16].

Female gender has been considered as a risk factor for HAEC [28], however, another study did not support this association [29]. Although univariate analysis showed that gender tended to affect the HAEC frequency after pull-through (Table 3), however, multivariate analysis did not (Table 6). Further studies with a larger sample size are necessary to confirm the association between gender and HAEC.

During interpretation of our study, the following limitations should be noted: this study was a retrospective medical records review over multiple years with a small sample size in each group partly because of the heterogeneity of the pull-through procedures selected according to the attending physician preference. While the incidence of HAEC and long-term continence are often related (the more continent, the higher frequency of HAEC is estimated), however, our study did not determine the long-term continence, becoming one of our study’s limitations.

## Conclusions

The lower cut-off of ≥4 might increase the possibility to diagnose HAEC, particularly the mild cases. The incidence of HAEC after definitive surgery was not associated with any risk factors in our cohort patients. Further multicenter studies with a larger sample size are necessary to confirm our results.

## Supplementary information

**Additional file 1 Supplement Table 1.** Association between patients’ characteristics and HAEC (cut-off ≥10) following Duhamel and Soave surgeries.

**Additional file 2 Supplement Table 2.** Risk factors for HAEC (cut-off ≥10) following Duhamel and Soave surgeries.

**Additional file 3 Supplement Table 3.** Logistic regression of risk factors and HAEC (cut-off ≥10) after pull-through in our institution.

## Data Availability

All data generated or analyzed during this study are included in the submission. The raw data are available from the corresponding author on reasonable request.

## Abbreviations

HAEC: Hirschsprung-associated enterocolitis
HSCR: Hirschsprung disease
