# Supplementary material for: Postoperative enterocolitis assessment using two different cut-off values in the HAEC score in Hirschsprung patients undergoing Duhamel and Soave pull-through
Source: BMC Pediatr. 2020 Oct 2;20:457. doi: 10.1186/s12887-020-02360-x (PMC7531158; doi:10.1186/s12887-020-02360-x)
Supplement: Supplementary file 1 — Additional file 1 Supplement Table 1. Association between patients’ characteristics and HAEC (cut-off ≥10) following Duhamel and Soave surgeries. [file 12887_2020_2360_MOESM1_ESM.docx]

**Supplement Table 1.** Association between patients’ characteristics and HAEC (cut-off ≥10) following Duhamel and Soave surgeries.

|  | **Sex** | | ***p*-value** | **OR (95% CI)** | **Aganglionosis type** | | ***p*-value** | **OR (95% CI)** |
| --- | --- | --- | --- | --- | --- | --- | --- | --- |
|  | **Male** | **Female** |  |  | **Short-segment** | **Long-segment** |  |  |
| Duhamel HAEC (n, %) | 7/8 (87.5) | 1/8 (12.5) | 0.29 | 3.3 (0.4-30.9) | 7/8 (87.5) | 1/8 (12.5) | 0.80 | 0.7 (0.1-13.5) |
| Soave  HAEC (n, %) | 5/6 (83) | 1/6 (17) | 0.29 | 2.9 (0.5-17.1) | 5/6 (83) | 1/6 (17) | 0.62 | 1.8 (0.2-17.2) |

*, *p*-values were calculated using Fisher Exact or Chi-square tests; CI, confidence interval; HAEC, Hirschsprung-associated enterocolitis; OR, odds ratio
