# Supplementary material for: Postoperative enterocolitis assessment using two different cut-off values in the HAEC score in Hirschsprung patients undergoing Duhamel and Soave pull-through
Source: BMC Pediatr. 2020 Oct 2;20:457. doi: 10.1186/s12887-020-02360-x (PMC7531158; doi:10.1186/s12887-020-02360-x)
Supplement: Supplementary file 2 — Additional file 2 Supplement Table 2. Risk factors for HAEC (cut-off ≥10) following Duhamel and Soave surgeries. [file 12887_2020_2360_MOESM2_ESM.docx]

**Supplement Table 2.** Risk factors for HAEC (cut-off ≥10) following Duhamel and Soave surgeries.

|  | **Mothers’ age at childbirth (years)** | | ***p**** | **OR (95% CI)** | **Gestational age** | | ***p**** | **OR (95% CI)** | **Maternal educational level** | | ***p**** | **OR (95% CI)** |
| --- | --- | --- | --- | --- | --- | --- | --- | --- | --- | --- | --- | --- |
|  | **≤35** | **>35** |  |  | **Preterm** | **Full-term** |  |  | **No education-elementary** | **Junior, high school - bachelor** |  |  |
| Duhamel HAEC (n, %) | 5/8 (62.5) | 3/8 (37.5) | 0.39 | 0.5 (0.1-2.6) | 1/8 (12.5) | 7/8 (87.5) | 0.13 | 12.6 (0.5-341) | 2/8 (25) | 6/8 (75) | 0.88 | 1.1 (0.2-7.0) |
| Soave  HAEC (n, %) | 6/6 (100) | 0 | 0.46 | 3.1 (0.2-61.2) | 1/6 (7) | 5/6 (83) | 0.33 | 3.6 (0.3-47.3) | 1/6 (7) | 5/6 (83) | 0.35 | 0.3 (0.04-3.2) |

*, *p*-values was calculated using Fisher-Exact test; HAEC, Hirschsprung-associated enterocolitis; OR, odds ratio; CI, confidence interval
