# Supplementary material for: Postoperative enterocolitis assessment using two different cut-off values in the HAEC score in Hirschsprung patients undergoing Duhamel and Soave pull-through
Source: BMC Pediatr. 2020 Oct 2;20:457. doi: 10.1186/s12887-020-02360-x (PMC7531158; doi:10.1186/s12887-020-02360-x)
Supplement: Supplementary file 3 — Additional file 3 Supplement Table 3. Logistic regression of risk factors and HAEC (cut-off ≥10) after pull-through in our institution. [file 12887_2020_2360_MOESM3_ESM.docx]

**Supplement Table 3.** Logistic regression of risk factors and HAEC (cut-off ≥10) after pull-through in our institution.

|  | **Sex** | | **Aganglionosis type** | | **Mother’s age at childbirth** | | **Gestational age** | | **Maternal educational level** | |
| --- | --- | --- | --- | --- | --- | --- | --- | --- | --- | --- |
|  | *p** | OR (95% CI) | *p** | OR (95% CI) | *p** | OR (95% CI) | *p** | OR (95% CI) | *p** | OR (95% CI) |
| Duhamel | 0.99 | - | 0.72 | 0.6 (0.1-8.3) | 0.18 | 3.7 (0.5-26) | 0.99 | - | 0.56 | 0.5 (0.1-4.5) |
| Soave | 0.84 | 0.8 (0.1-9.6) | 0.82 | 0.8 (0.1-8.2) | 0.99 | - | 0.42 | 0.3 (0.02-5.1) | 0.42 | 2.6 (0.3-27) |

*, *p*-values was calculated using logistic regression test; CI, confidence interval; HAEC, Hirschsprung-associated enterocolitis; OR, odds ratio
